# *Helicobacter pylori* Infection Increased Anti-dsDNA and Enhanced Lupus Severity in Symptomatic FcγRIIb-Deficient Lupus Mice

**DOI:** 10.3389/fmicb.2018.01488

**Published:** 2018-07-06

**Authors:** Saowapha Surawut, Wimonrat Panpetch, Jiradej Makjaroen, Pattarin Tangtanatakul, Arthid Thim-Uam, Jutamas Wongphoom, Somying Tumwasorn, Asada Leelahavanichkul

**Affiliations:** ^1^Medical Microbiology Interdisciplinary Program, Graduate School, Chulalongkorn University, Bangkok, Thailand; ^2^Department of Microbiology, Faculty of Medicine, Chulalongkorn University, Bangkok, Thailand; ^3^Inter-Department Program of Biomedical Sciences, Faculty of Graduate, Chulalongkorn University, Bangkok, Thailand; ^4^Division of Pathology, Faculty of Medicine, Chulalongkorn University, Bangkok, Thailand; ^5^Center of Excellence in Immunology and Immune-mediated Diseases, Department of Microbiology, Faculty of Medicine Chulalongkorn University, Bangkok, Thailand

**Keywords:** *Helicobacter pylori*, fcγrIIb-deficient mice, lupus, susceptibility, murine model

## Abstract

The defect on Fc gamma receptor IIb (FcγRIIb), the only inhibitory FcγR, has been identified as one of the genetic factors increasing susceptibility to lupus. The prevalence of *Helicobacter pylori* (HP) and FcγRIIb dysfunction-polymorphisms are high among Asians, and their co-existence is possible. Unfortunately, the influence of HP against lupus progression in patients with lupus is still controversial. In this study, the *interactions* between these conditions were tested with HP infection in 24-week-old FcγRIIb-/- mice (symptomatic lupus). HP induced failure to thrive, increased stomach bacterial burdens and stomach injury (histology and cytokines) in both wild-type and FcγRIIb-/- mice. While the severity of HP infection, as determined by these parameters, was not different between both strains, antibodies production (anti-HP, anti-dsDNA and serum gammaglobulin) were higher in FcγRIIb-/- mice compared to wild-type. Accordingly, HP infection also accelerated the severity of lupus as determined by proteinuria, serum creatinine, serum cytokines, renal histology, and renal immune complex deposition. Although HP increased serum cytokines in both wild-type and FcγRIIb-/- mice, the levels were higher in FcγRIIb-/- mice. As such, HP also increased spleen weight and induced several splenic immune cells responsible for antibody productions (activated B cell, plasma cell and follicular helper T cell) in FcγRIIb-/- mice, but not in wild-type. These data describe the different systemic responses against localized HP infection from diverse host genetic background. In conclusion, the mutual interactions between HP and lupus manifestations of FcγRIIb-/-mice were demonstrated in this study. With the prominent immune responses from the loss of inhibitory signaling in FcγRIIb-/- mice, HP infection in these mice induced intense chronic inflammation, increased antibody production, and enhanced lupus severity. Thus, the increased systemic inflammatory responses due to localized HP inducing gastritis in some patients with lupus may enhance lupus progression. More studies are needed.

## Introduction

*Helicobacter pylori* (HP), microaerophilic, spiral-shaped gram-negative bacteria, are organisms that can survive in the highly acidic stomach environment, and are known to cause chronic gastric inflammation and cancer (Mahachai et al., 2016). The infection is very common among Asians, with a prevalence rate of up to 50–80% in some countries (Thirumurthi and Graham, 2012; Xie and Lu, 2015). Interestingly, eradication of HP in some patients with associated autoimmune diseases leads to long-term remission of the autoimmune disease (Fujimura et al., 2005; Kuwana, 2014). Moreover, HP infection down-regulates the expression of Fc gamma receptor IIb (FcγRIIb), the only inhibitory FcγR (Bolland and Ravetch, 2000) on circulating monocyte of patients with autoimmune diseases (Asahi et al., 2008; Wu et al., 2012). As Fcγ receptors (FcγR) is the immunoglobulin superfamily that contributes to the protective functions, in part, by inducing phagocytosis of opsonized microbes, loss of the inhibitory FcγR results in effective organism control but enhances the risk of autoimmune diseases (Ravetch and Bolland, 2001). Although HP infection has shown a protective effect on the development of lupus in a case control study, especially among African-American patients, the relationship of lupus-HP is still intriguing (Sawalha et al., 2004; Hasni et al., 2011).

Inadvertently, FcγRIIb dysfunction polymorphisms are common in Asia (Chu et al., 2004), partially due to the genetic pressure from malarial infection (Clatworthy et al., 2007). Although FcγRIIb dysfunction protects against malaria, the insufficient inhibitory signaling increases the risk of autoimmune activation. Indeed, the association between FcγRIIb polymorphisms and systemic lupus erythematosus (lupus) in patients has been reported (Tsuchiya and Kyogoku, 2005). Both FcγRIIb dysfunction polymorphisms and HP infection are common in the Asian population (Smith and Clatworthy, 2010; Hooi et al., 2017). While FcγRIIb loss-of-function is associated with lupus (Siriboonrit et al., 2003; Tsuchiya and Kyogoku, 2005; Jakes et al., 2012), HP infection has been associated with other autoimmune diseases such as immune thrombocytopenic purpura and membranous nephropathy (Hasni et al., 2011). As chronic inflammation accelerates lupus (Hasni et al., 2011) and the co-existence of FcγRIIb dysfunction polymorphisms with HP infection are possible, information on the responses of FcγRIIb-/- mice to of HP infection in patients with lupus. Thus this study tested HP infection in FcγRIIb-/- condition, *in vivo* and *in vitro*.

## Materials and Methods

### Animal

FcγRIIb-/- mice on C57BL/6 background were kindly provided by Dr. Silvia Bolland (NIAID, NIH, Maryland, United States). Wild-type female C57BL/6 mice were purchased from the National Laboratory Animal Center in Nakhon Pathom Province, Thailand. The animal protocols, as per NIH criteria, were approved by the Faculty of Medicine, Chulalongkorn University. Due to lupus manifestations’ age-dependency, female symptomatic lupus (24-week-old with kidney injury) mice or age-matched wild-type control groups were used. Serum samples were collected through tail-vein nicking and through cardiac puncture at sacrifice for time-course analysis. Mice were sacrificed with cardiac puncture under isoflurane anesthesia and internal organs were collected, fixed in 10% formalin and embedded in paraffin. Staining in 4-μm sections with haematoxylin and eosin color (H&E) were used for further evaluation.

### *Helicobacter pylori* Administration Model

HP ATCC 43504 (ATCC, Manassas, VA, United States) was cultured on supplemented Columbia agar (Oxoid, Hampshire, United Kingdom) under microaerophilic conditions (6–12% O_2_, 5–8% CO_2_) at 37°C for 48 h before use. The mouse model for HP infection was modified from a previous study (Konturek et al., 1999). Briefly, HP at 2 × 10^9^ CFU/ml in 0.5 ml or phosphate buffer solution (PBS) control were orally administered twice daily for 2 weeks and once daily 3 weeks after. Mice were sacrificed at 1 week after the last administration of HP. Mouse blood was centrifuged and serum was kept at -80°C until analysis. Stomach was divided longitudinally through the greater and lesser curvature into several parts, washed with PBS, weighed and used to test HP burdens (i) by urease test, polymerase chain reaction (PCR) and direct culture, (ii), histopathology (fixed in 10% formaldehyde) and (iii) cytokine analysis from gastric tissue.

### Urease Test, a Semi-Quantitative Analysis of Gastric *Helicobacter pylori* Burden

The principle of urease test is based on HP’s urease enzyme production (Midolo and Marshall, 2000). Urease enzyme splits urea metabolites into ammonia and carbon dioxide, and ammonia alkalinizes the culture media (the media color turns from yellow to pink). The stomach specimens (one-fourth of the total stomach tissue; 50 mg) were minced and directly put onto urea agar slant, and incubated at 37°C. The media color was observed at 24 h after incubation. As the color media alteration starts from the top to the bottom (**Figure 3**), the ratio of the pink color to the total depth of the media was used as a semi-quantitative measurement of HP burden.

### Polymerase Chain Reaction (PCR), a Quantitative Analysis of Gastric *Helicobacter pylori* Burden

Quantitative real-time PCR of HP from gastric tissue were performed as previously described (He et al., 2002). In short, genomic DNA was extracted by High Pure PCR Template Preparation Kit (Roche, United States), and quantified by spectrophotometry (NanoDrop^TM^, Thermo Fisher Scientific, United States). The primers of *UREC* gene-fragment were HP-FOR (5′- TTATCGGTAAAGACACCAGAAA -3′) and HP-REV (5′- ATCACAGCGCATGTCTTC -3′). The amplification product was 132 bp and the genome size of HP ATCC 43504 (also designated HP CCUG 17874) was 1,615,763 bp (Clancy et al., 2012). Bacterial genome is approximately 1.06 × 10^9^ g/mol and contains 6.02 × 10^23^ molecules/mol. One bacterium corresponds to 1.8 fg of DNA. The constructive of standard curve was created by the LightCycler software using 10-fold serial dilution (3.6 fg-360 pg) per 5 μl of HP DNA, with bacterial concentrations ranging from of 2 to 2 × 10^5^ bacteria. The profiling standard curve was indicated as a graph of crossing point (Cp) vs. bacterial number (CFUs). HP quantification was calculated by the standard curve and shown in bacterial number (CFUs).

### Direct Culture, a Quantitative Analysis of Gastric *Helicobacter pylori* Burden

As an additional method, burdens of HP from gastric tissue were performed in a selective media (Skene et al., 2007). In brief, gastric samples were weighed, homogenized in 1 ml PBS and serially plated onto the supplemented Columbia agar (Oxoid, Hampshire, United Kingdom) with 10 mg/l vancomycin, 5 mg/l amphotericin B under microaerophilic conditions at 37°C for 48 h before bacterial enumeration.

### Stomach Histology and Anti-*Helicobacter pylori* Antibody

Inflammatory response in stomach was scored by 2 blinded observers according to the Sydney Scoring System at 200 × magnification in 10 randomly selected fields of each sample (Dixon et al., 1996). The scoring system was determined by leukocyte mucosal infiltration with the following criteria; Score 0, normal; Score 1, mild; Score 2, moderate; Score 3 marked histopathological changes. In addition, serum anti-*H. pylori* IgG antibody (anti-HP) was measured by ELISA (My BioSource Inc, SanDiego, CA, United States).

### Cytokines Measurements in Gastric Tissues and Serum

Cytokine in gastric tissue was measured using the same method as in a previous publication (Ferrero et al., 2008). Briefly, gastric tissues were homogenized (Ultra-Turrax homergenizer, IKA, Staufen, Germany) in 500 μl of PBS containing protease inhibitor, centrifuged at 12,000 × g for 10 min at 4°C. The supernatant was collected and stored at -80°C until analyzed. Quantikine ELISA (ReproTech, NJ, United States) was used to measure CXC chemokines (MIP-2 and KC) and pro-inflammatory cytokines (IL-1β and TNF-α) in the supernatant and the serum.

### Lupus Characteristics (Anti-dsDNA, Proteinuria, Serum Creatinine, and Renal Histology)

Lupus characteristics were analyzed as per the protocol in previous publication (Surawut et al., 2017). In brief, serum anti-dsDNA measurement was based on the ELISA assay of calf DNA (Invitrogen, Carlsbad, CA, United States) coated on 96-well plates (Surawut et al., 2017). Urine protein creatinine index (UPCI), a representative of 24 h proteinuria, was determined by the equation; spot urine protein/spot urine creatinine (Leelahavanichkul et al., 2010). Urine protein was measured by bradford protein assay. Urine and serum creatinine levels were detected by QuantiChrom Creatinine Assay (DICT-500, BioAssay, and Hayward, CA, United States). For renal histology, kidneys were fixed in 10% formalin, paraffin embedded, and samples at 4 mm thickness were stained with Periodic acid–Schiff (PAS) reagent (Sigma-Aldrich) for the modified semi-qualitative assessment (Leelahavanichkul et al., 2010). Percentage of severe glomerular damage as determined by mesangial expansion > 60% of glomerular area or glomeruli with crescent formation was estimated at 400 × magnification to represent the severity of glomerular injury. Renal interstitial injury was estimated at 200 × magnification on 10 randomly selected fields for each kidney by the observation of the area of cellular infiltration in each field with the following semi-quantitative criteria: 0, area < 5%; 1, areas 5–15%; 2, area 15–30%; 3, area > 30–60%; 4, area > 60%. In addition, the immune complex deposition in renal tissue was detected by immunofluorescence following a standard protocol. In brief, the frozen section was fixed with acetone for 10 min and blocked with 1% bovine serum albumin (BSA) in PBS for 1 h at room temperature. The section was then stained with anti-Fc IgG for 1 h followed by IgG-Alexa-488 (secondary antibody). Then 300 nm of DAPI was added and incubated for 5 min in the dark.

### An Analysis of Serum Gamma Globulin

For the evaluation of antibody responses, mouse serum was analyzed for total immunoglobulin by capillary protein electrophoresis (MINICAP-2 Sebia, Evry Cedex, France). The percentage of protein in the gamma zone of protein electrophoresis was converted into total immunoglobulin level by multiplying the ratio of protein at the gamma zone by serum total protein. Serum total protein and urine protein were measured by Bradford protein assay.

### Flow Cytometry Analysis of Spleen

Flow cytometric analysis was performed following a standard protocol. In brief, spleens were minced in supplemented RPMI-1640 (Roswell Park Memorial Institute media), and the cells were centrifuged at 300 g for 5 min at 4°C. Red blood cells were removed using lysis buffer (ACK buffer: NH4Cl, KHCO_3_ and EDTA) and the splenocytes were washed twice in supplemented RPMI-1640. Subsequently, the splenocytes were resuspended in staining buffer (0.5% BSA and 10% FBS in PBS), and then were stained with fluorochrome-conjugated antibodies against different mouse immune cells including; CD19, CD80, CD138, CD3, CD4, CXCR5, B220, CD19, GL-7 and F4/80 (BioLegend). All stained cells were analysed by flow cytometry using BD LSR-II (BD Biosciences) and data analysis by FlowJo software (version 10).

### Macrophages Phagocytosis and Macrophage Killing Activity

Macrophages were derived from the bone marrow (BM) (Ondee et al., 2017) and phagocytosis assay was performed following an established protocol (Chmiela et al., 1997; Miliukene et al., 2007). In short, 1 × 10^9^ cell/ml heat-killed HP (60°C for 30 min) was incubated with 100 μg/ml fluorescein isothiocyanate (FITC) (Sigma, United States) in PBS at 35°C for 30 min for FITC-labeling. This was then mixed with activated macrophages at multiplicity of infection (MOI) 500:1 (HP-FITC: macrophage) in DMEM complete media with 5% mouse normal serum (as opsonin) in 96-well polystyrene tissue culture plates. Of note, the high MOI was necessary for the adequate fluorescent intensity for the detection of phagocytosis activity. The incubation with 10 ng/ml IFN-γ (Biolegend, United States) for 17 h followed by 100 ng/ml of lipopolysaccharide (LPS; Sigma, United States) for 24 h was performed for macrophage activation. Supernatant IL-12p70 was measured by ELISA (eBioscience, United States) to support the activated-state of macrophage.

Macrophages and HP were allowed for phagocytosis for 0.5, 1, and 2 h. At each time point, all media was removed and 100 μl/well of 0.2% trypan blue in PBS was added to quench extracellular FITC labeled-bacteria. Phagocytosis activity was determined by the detection of intracellular bacteria with fluorescent intensity read at 492 nm excitation and 518 nm emission wavelengths. On the other hand, the killing assay was performed as previously published (Keep et al., 2010). In brief, live HP to macrophage at MOI 500:1, as described above, was allowed for phagocytosis for 0.5 h. Then, the media and extracellular bacteria were removed, 100 μg/ml gentamycin was added for 1 h at 37°C, 5% CO_2_ to eliminate the remaining extracellular bacteria. Cells were washed with PBS 5 times, the final wash was plated in Columbia agar (Oxoid, United Kingdom) to ensure that no extracellular bacteria remained after washing. Then the remaining HP-phagocytized cells were used to determine the intracellular bacteria at 0.5 h phagocytosis, while some of the wells with HP-phagocytized cells were further incubated for 2 and 6 h after extracellular bacteria removal process before bacterial determination. For intracellular bacterial determination, 200 μl/well of 0.1% saponin was added for 15 min at 37°C, 5% CO_2_ to release intracellular bacteria, serially diluted and plated on Columbia agar (Oxoid, United Kingdom) incubated under micro-aerophilic conditions at 37°C up to 5 days for colony enumeration. The macrophage killing activity was determined by intracellular proliferation rate at each time-point by the ratio of colony forming unit (CFU) of bacteria at the specific time-point divided by CFU at 0.5 h phagocytosis.

Since immune complex (IC) can affect phagocytosis and killing activity of macrophage, the functions with and without IC treated conditions were tests. The ICs were generated as described previously (Ding et al., 2015). Briefly, mouse IgG1 anti-ovalbumin (anti-OVA; Sigma-Aldrich) was mixed with ovalbumin (OVA; Sigma-Aldrich) followed by incubation at room temperature for 30 min to allow IC formation. Then the ICs were challenged with macrophages for 24 h before performing phagocytosis and killing assay. Macrophages without ICs activation was used as a control condition. In addition, to test if serum of symptomatic FcγRIIb-/- mice influenced macrophage functions, 10% mouse serum (in DMEM) from FcγRIIb-/- (symptomatic lupus) or wild-type (control) was incubated with macrophage for 24 h before performing phagocytosis and killing activity assay.

### Statistical Analysis

Mean ± standard error (SE) was used for data presentation. Unpaired Student’s *t*-test or one-way analysis of variance (ANOVA) with Tukey’s comparison test was used for the analysis of experiments with groups 2 and 3, respectively, The repeated measures ANOVA with Bonferroni *post hoc* analysis was used for the analysis of data with several time-points. *P-*values < 0.05 were considered statistically significant. SPSS 11.5 software (SPSS Inc., Chicago, IL, United States) was used for all statistical analysis.

## Results

### The Prominent Anti-*Helicobacter pylori*, With Similar Disease Severity to Wild Type, in *Helicobacter pylori* Infection of Symptomatic Lupus FcγRIIb Deficient-Mice

**FIGURE 1:**
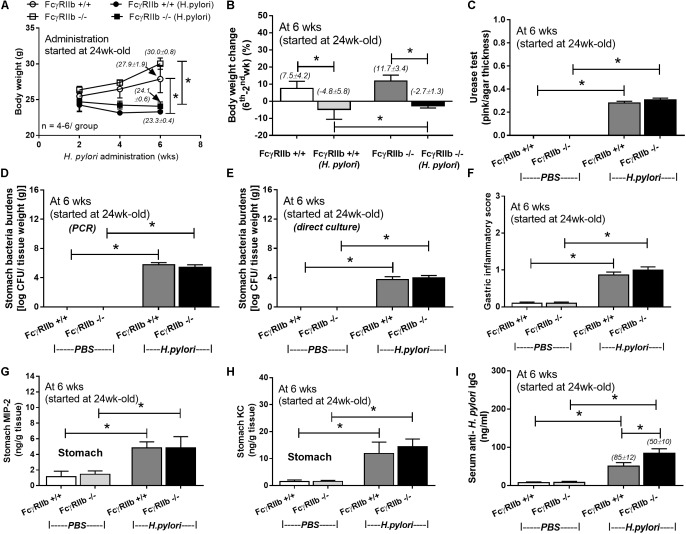
Severity of *H. pylori* (HP) infection in symptomatic lupus (24-week-old) (FcγRIIb-/-) and wild-type (FcγRIIb +/+) mice as determined by body weight **(A)**, body weight alteration **(B)**, stomach bacterial burdens by the semi-quantitative scoring of urease test, polymerase chain reaction (PCR) and the direct culture on selective media (direct culture) **(C–E)**, gastric inflammatory score **(F)**, gastric cytokines (MIP-2 and KC) **(G,H)**, and serum anti-HP IgG antibody **(I)** (*n* = 5–7/group) were demonstrated. MIP-2; Macrophage Inflammatory Protein-2, KC; Keratinocyte Chemoattractant; ^∗^*p* < 0.05.

**FIGURE 2:**
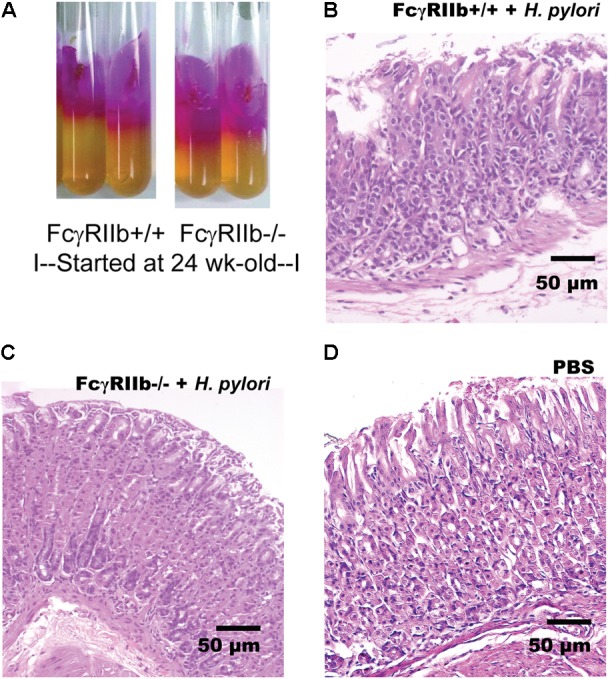
Representative figures of urease test from gastric tissue of mice with *H. pylori* (HP) infection in the wild-type (FcγRIIb +/+) and lupus (FcγRIIb-/-) mice **(A)** were demonstrated. Gastric histopathology in wild-type and FcγRIIb-/- mice with HP infection **(B,C)** and wild-type with phosphate buffer solution (PBS) gavage **(D)** are shown. (Of note, gastric histology of FcγRIIb-/- mice with PBS was not demonstrated due to the non-difference from wild-type with PBS.)

Lupus characteristics as determined by serum creatinine, proteinuria, and anti-dsDNA in FcγRIIb-/- mice after 24-week-old is previously described (Ondee et al., 2017; Surawut et al., 2017). Hence, HP administration in 24-week-old mice represents HP infection in patients with symptomatic lupus. Indeed, HP infection caused significant weight loss in both wild-type and FcγRIIb-/- mice (**Figures 1A,B**) and the wild-type demonstrated a little bit more prominent weight loss. In addition, similar disease severity between both strains were demonstrated with (i) bacterial burdens in stomach with semi-quantitative assay (urease test) (**Figures 1C**, **2A**) and quantitative methods (real-time PCR and direct culture from stomach tissue) (**Figures 1D,E**), (ii) histological inflammation in stomach (**Figures 1F**, **2B–D**) and (iii) macrophage inflammatory protein-2 (MIP-2) and keratinocyte-derived cytokine (KC), the important chemokines of HP pathogenesis (Sgouras et al., 2005; De Filippo et al., 2008), in gastric-tissue (**Figures 1G,H**). Despite the similar severity of HP infection between both strains, FcγRIIb-/- mice had the higher serum anti-HP IgG than wild-type (**Figure 1I**).

### *Helicobacter pylori* Infection Enhanced Anti-dsDNA Levels and Clinical Characteristics of Lupus in 24-Week-Old FcγRIIb Deficient-mice

**FIGURE 3:**
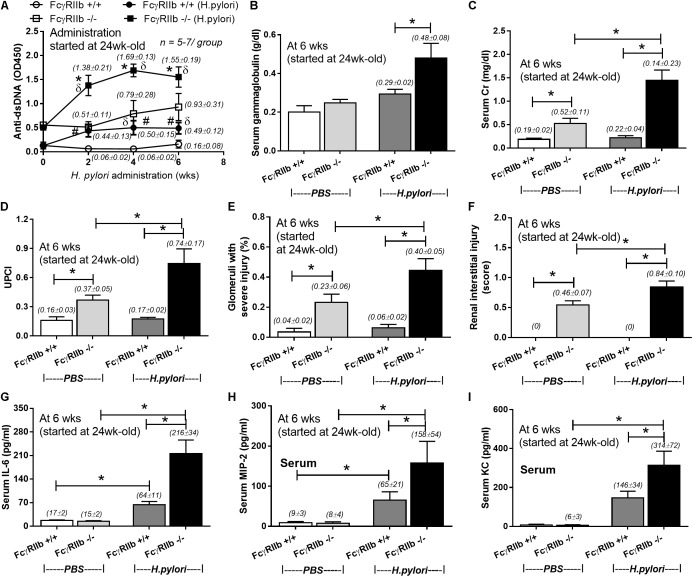
Severity of lupus in FcγRIIb-/- mice and wild-type (FcγRIIb +/+) with and without *H. pylori* (HP) infection as determined by anti-dsDNA **(A)**; serum gammaglobulin **(B)**; serum creatinine **(C)**; urine protein creatinine index (UPCI) **(D)**; renal injury score of glomerular and tubular lesion **(E,F)**; and serum cytokines (IL-6, MIP-2 and KC) **(G–I)** were demonstrated (*n* = 5–7/ time-point and 5–7/group). MIP-2; Macrophage Inflammatory Protein-2, KC; Keratinocyte Chemoattractant; ^∗^*p* < 0.05.

**FIGURE 4:**
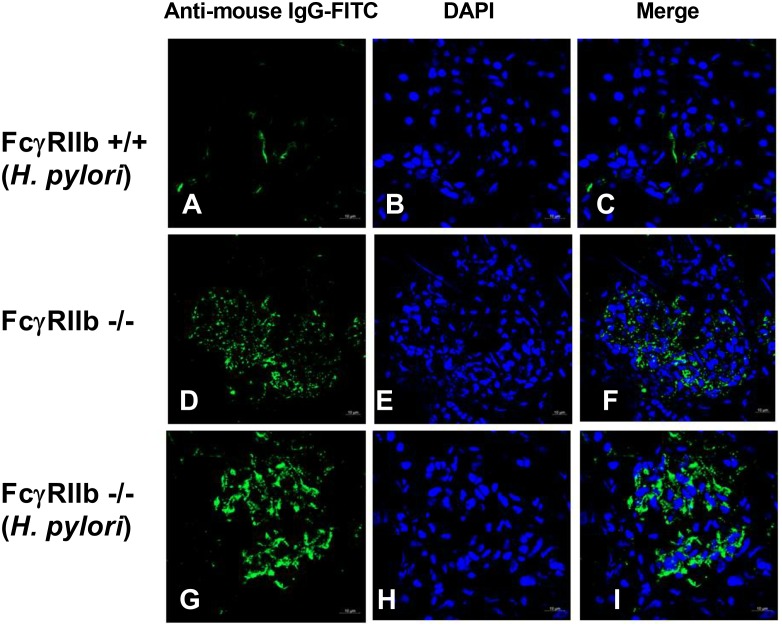
Representative immunofluorescence images from glomeruli of wild-type (FcγRIIb +/+) with *H. pylori* (HP) **(A–C)**, FcγRIIb-/- mice with control phosphate buffer solution (PBS) gavage **(D–F)**, and FcγRIIb-/- mice with *H. pylori* administration **(G–I)** were demonstrated. Original magnification 600×; goat anti-mouse IgG with Fluorescein isothiocyanate (FITC) (green) and DAPI (blue) were used for the identification of Fc portion of the immune complex deposition and nucleus, respectively, (Of note, the glomerulus of wild-type mice with PBS was not demonstrated due to the non-difference from wild-type with HP.)

**FIGURE 5:**
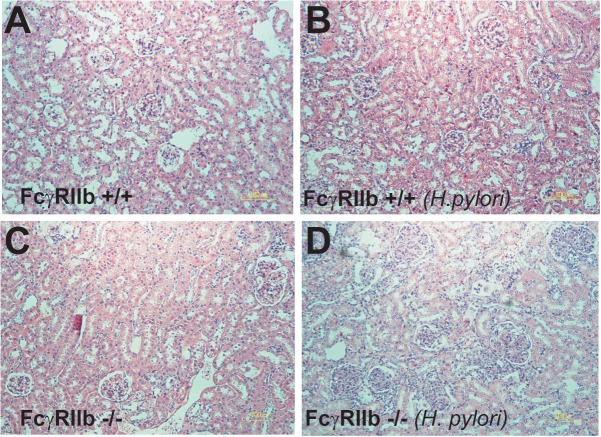
Representative figures of renal histology of wild-type (FcγRIIb +/+) and FcγRIIb-/- mice with phosphate buffer solution (PBS) gavage **(A,B)** and *H. pylori* administration **(C,D)** were demonstrated. Original magnification 400 ×.

HP infection has been shown to enhance auto-antibodies (Yamanishi et al., 2006; Hasni et al., 2011), thus this study specifically looked at anti-dsDNA levels, a specific auto-antibody of lupus, which also helps determine disease severity. Indeed, HP induced anti-dsDNA in wild-types and accelerated anti-dsDNA level in FcγRIIb-/- (**Figure 3A**). Although HP induced anti-dsDNA in both mouse strains, anti-dsDNA levels were more prominent in FcγRIIb-/- group (**Figure 3A**), implying the autoimmune inducibility of FcγRIIb-/- mice. As most of the serum protein in gamma zone of serum protein electrophoresis analysis is immunoglobulin, serum gamma globulin is measured as a representative of total immunoglobulin. Interestingly, HP infection enhanced immunoglobulin production in both strains but more predominantly in FcγRIIb-/- mice (**Figure 3B** and **Supplementary Figure S1**). This is in concordance with the higher level of serum anti-HP and anti-dsDNA in FcγRIIb-/- mice (**Figures 1I**, **3A**). In addition, there was prominent immunoglobulin deposition in the glomeruli of FcγRIIb-/- mice (**Figure 4**) with the enhanced severity of lupus nephritis as demonstrated with serum creatinine, urine protein (urine protein creatinine index; UPCI), renal histology and serum cytokines (IL-6, MIP-2 and KC; the representatives of systemic inflammatory responses) (**Figures 3C–H**, **5**). HP induced chemokine responses both locally (gastric tissue) (**Figures 1H,I**) and systemically (**Figures 3H,I**). With HP infection, gastric cytokines were not different between wild-type and FcγRIIb-/- mice but serum cytokines were more prominent in the FcγRIIb-/- group.

### Enhanced Immune Cells in the Spleen of FcγRIIb Deficient-Mice With *Helicobacter pylori* Infection

**FIGURE 6:**
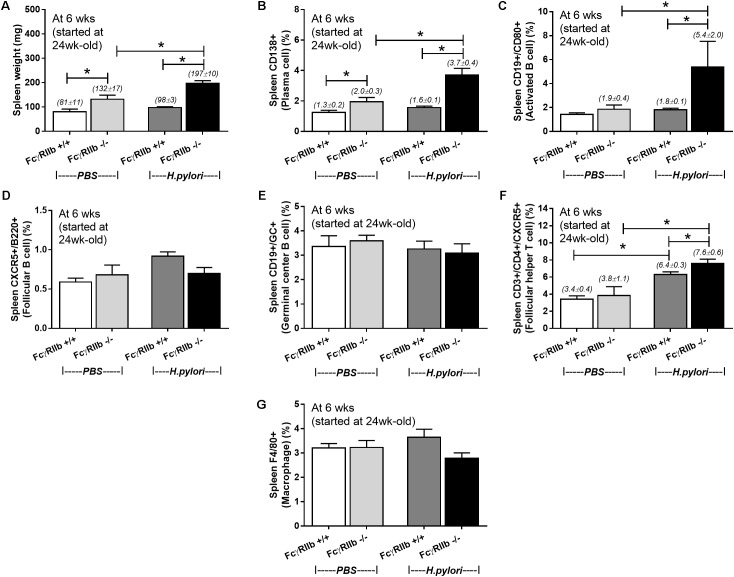
Spleen weight (*n* = 5/group) **(A)** and the quantitative flow-cytometric analysis of splenocytes from wild-type (FcγRIIb +/+) and FcγRIIb-/- mice with phosphate buffer solution (PBS) gavage or *H. pylori* administration in the spleen plasma cell (CD138 +) **(B)**, spleen activated B cell (CD19 + and CD80 +) **(C)**, spleen germinal center B cell (CD19 + GC +) **(D)**, spleen follicular B cell (CXCR5 + and B220 +) **(E)**, spleen follicular helper T cell (CD3 + and CD4 + and CXCR5 +) **(F)** and spleen macrophage (F4/80 +) **(G)** were demonstrated. (*n* = 5–8/group); ^∗^*p* < 0.05

**FIGURE 7:**
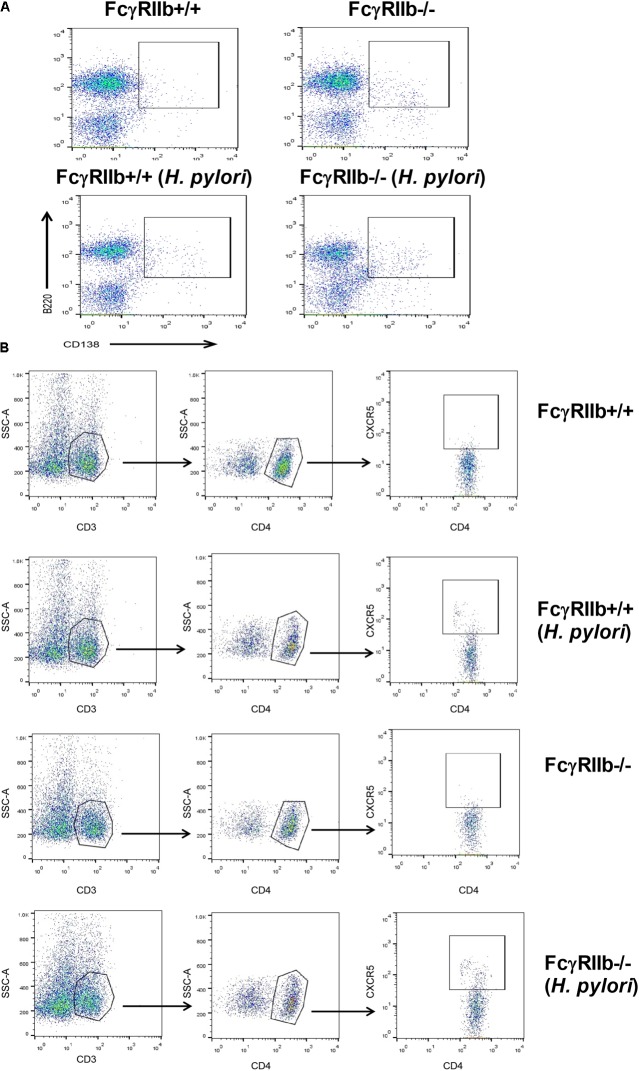
The representatives of flow-cytometric analysis were demonstrated; spleen plasma cell **(A)** and spleen follicular helper T cell **(B)**.

Due to the prominent immunoglobulin production in FcγRIIb-/- mice with HP infection, lymphoid organs in these mice were examined. It is surprising that there was non-difference in gastric weight and mucosal associated lymphoid tissue morphology between wild-type and FcγRIIb-/- mice with or without HP infection (data not shown). Spleen weight among these mice was different. Spleen weight without HP infection of FcγRIIb-/- mice was higher than wild-type and the infection enhanced FcγRIIb-/- spleen weight but not wild-type spleen (**Figure 6A**). Thus, we examined splenocyte by flow cytometry analysis (**Figures 6B–G**, **7**). Without HP, plasma cell (CD138 +) was the only cell population that was different between wild-type and FcγRIIb-/- mice (**Figure 6B**). In FcγRIIb-/- mice with HP infection, there was an increase in plasma cells, activated B cells (CD19 + and CD80 +) and follicular helper T cells (CD3 + , CD4 + and CXCR5 +) but not follicular B cells (CXCR5 + and B220 +), germinal center B cells (CD19 + and GC +) and macrophages (F4/80 +) in comparison with FcγRIIb-/- without HP or wild-type with HP (**Figures 6B–G**). Only follicular helper T cells were increased in wild-type with HP compared to wild-type without HP infection (**Figure 6F**). This data supports the hyper-immune response of FcγRIIb-/- against HP compared with wild-type.

### The Hyper-Responsiveness of FcγRIIb-/- Macrophages to *Helicobacter pylori* Activation and the Loss of Response With Immune Complex Treatment

**FIGURE 8:**
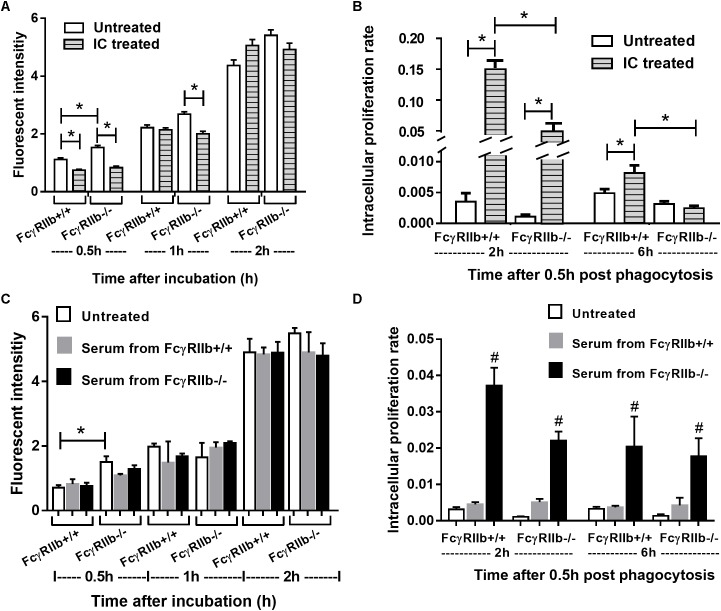
Phagocytosis and killing activity of macrophage from wild-type (FcγRIIb +/+) and FcγRIIb-/- mice in response against *H. pylori* and these functions after incubation with immune complex incubation **(A,B)** or mouse serum from wild-type and FcγRIIb-/- mice **(C,D)** were demonstrated (experiments were performed in triplicate). ^∗^*p* < 0.05; ^#^*p* < 0.05 vs. untreated or treated with serum from FcγRIIb-/- mice.

We examined macrophage functions, *in vitro*, as a pilot experiment because (i) the influence of macrophage against HP in lupus and wild-type might be different, (ii) the possible effect of high circulating immune complex against macrophage functions (Bolland and Ravetch, 2000; Clatworthy et al., 2007; Surawut et al., 2017) and (iii) the lack of data on FcγRIIb-/- macrophage against HP. Interestingly, FcγRIIb-/- macrophage showed more prominent phagocytosis than wild-type after 0.5 h of incubation, however, this was not the case after 1 or 2 h of incubation (**Figures 8A,C**). In addition, the IC incubation reduced phagocytosis at 0.5 h in both groups of macrophage and at 1 h in FcγRIIb-/- group (**Figure 8A**). Further, FcγRIIb-/- macrophage showed prominent killing activity over wild-type at both time-points (lower intracellular bacteria) (**Figures 8B,D**). After IC-incubation, the killing activity worsened in both strains of macrophage, but predominantly in the wild-type cells (**Figure 8B**). Interestingly, pre-treatment macrophage with serum from FcγRIIb-/- mice, but not wild-type serum, reduced macrophage killing activity without the influence on phagocytosis activity (**Figures 8C,D**). This implies that there are some macrophage-neutralizing factors in serum of symptomatic lupus mice.

## Discussion

Although a previous case-control study demonstrates low prevalence of HP infection in patients with lupus (Sawalha et al., 2004), there has been no other studies exploring the lupus-HP relationship. Despite the case-control study in that report, it is still unclear if the immune response of patients with lupus prone genes is effective against HP or HP infection is protective for lupus. Hence, we explored lupus-HP relationship in FcγRIIb-/- lupus mice in this study.

There was prominent antibody production but similar severity of HP gastritis in FcγRIIb-/- mice in comparison with wild-type. Enhanced immune responses and effective organism control due to the defect of FcγRIIb, the only inhibitory receptor of the FcγR family, is well-known (Bolland and Ravetch, 2000). Despite these benefits, the severity of HP infection, demonstrated by survival, bacterial burdens and gastric-cytokines, were similar between FcγRIIb-/- and wild-type. Surprisingly, anti-HP antibody was more prominent in FcγRIIb-/- mice. Despite the beneficial effects of anti-HP antibody against HP in wild-type mice (Kaparakis et al., 2008), the antibody in FcγRIIb-/- mice seemed to be less effective.

There was enhanced antibody production and lupus acceleration in FcγRIIb-/- mice with HP gastritis, a localized infection. There was not only the increase in anti-HP, a specific antibody against HP, but also anti-dsDNA and serum gamma immunoglobulin in FcγRIIb-/- mice with HP. Interestingly, it seems that the spleen, but not the gastric lymphoid tissue, was responsible for the prominent antibody production in FcγRIIb-/- with HP because of the increased weight of the spleen, not the stomach. HP infection induced several immune cells that associated with the antibody production process in the spleen, including activated B cell, plasma cell and follicular helper T-cell. In the wild-type with HP, anti-HP increased without spleen activation and serum immunoglobulin. Indeed, HP infection activated only gastric lymphoid organ and lymphocyte but not the spleen in the wild-type mice (Bussiere et al., 2006; Floch et al., 2015). The systemic response against HP (serum cytokines) was found only in FcγRIIb-/- mice but not wild-type supporting the hyper-inflammatory responses of FcγRIIb-/- mice. It is interesting to note that increased serum cytokines after HP infection is demonstrated in most of the studies on mouse model (Kodama et al., 2005) and juvenile patients (Khaiboullina et al., 2016) but not in other group of patients (Bayraktaroğlu et al., 2004). Although there is no data on the influence of increased systemic cytokines in patients with HP, our study implicated the importance of increased systemic cytokines in lupus with HP infection.

It is well known that chronic infection and inflammation initiates and accelerates lupus (Munoz et al., 2010; Esposito et al., 2014; Podolska et al., 2015; Rigante and Esposito, 2015) and the hyper-inflammatory responses in FcγRIIb-/- mice due to the inhibitory signaling defect (Clatworthy et al., 2007) might enhance this effect. Thus, increased antibody production in FcγRIIb-/- mice with HP might be due to the prominent systemic responses against HP infection. Perhaps, the persistent gastric inflammation from HP infection induces epitope spreading and/or bystander activation in FcγRIIb-/- mice resulting in the increased auto-antibody. As such, HP enhanced anti-dsDNA levels in both strains, but more predominantly in FcγRIIb-/-, supports the well-known HP-induced autoimmunity hypothesis (Hasni et al., 2011). In addition, the increased anti-dsDNA in FcγRIIb-/- mice also enhanced lupus severity, supporting its role in lupus pathogenesis (Giles and Boackle, 2013).

Regarding the increased anti-HP antibody (**Figure 1I**) and the prominent macrophage functions (Bolland and Ravetch, 2000; Clatworthy et al., 2007; Surawut et al., 2017) in FcγRIIb-/- mice, the lower bacterial burdens and the more severe gastritis were expected in these mice compared with wild-type. This is due to the attenuation property of anti-HP and the macrophage enhanced gastritis in macrophage depleted wild-type mice (Kaparakis et al., 2008). Surprisingly, bacterial burdens and gastritis severity were not different between the 2 groups. This suggests different influence of immune responses or different neutralizing factor of antibody between wild-type and lupus mice. Moreover, infection susceptibility and high circulating immune complex (CIC) in symptomatic lupus is well-known, thus the negative influence of CIC in lupus against infection is possible. Indeed, we demonstrated the inhibitory effect of IC and lupus mouse serum against macrophage phagocytosis and killing activity, *in vitro*, in both wild-type and FcγRIIb-/- macrophage which, at least in part, explained the infection susceptibility of lupus. Despite several macrophage-neutralizing factors (e.g., uremic toxins) in FcγRIIb-/- mouse serum, CIC might contribute some influences. More studies on this subject are necessary.

Several limitations of translation research should be considered before applying any clinical translation such as; (i) mouse is not the natural host of HP and repeated gavage of HP is different from the disease’s natural course, (ii) there are some different properties and expressions of FcγRIIb receptor between human and mouse (Bruhns, 2012; Hussain et al., 2015), (iii) unmeasured factors might also affect the model due to other FcγRIIb-/- characteristics, and (iv) the opsonin used for macrophage activity assays, *in vitro*, might not resemble the *in vivo* physiology.

## Conclusion

Prominence of anti-HP, anti-dsDNA and increased serum immunoglobulin despite the similar disease severity of HP infection in FcγRIIb-/- mice compared with wild-type were demonstrated. HP infection in FcγRIIb-/- mice enhanced systemic inflammation, induced antibody-producing immune cells in the spleen and enhanced lupus disease severity. Thus, the localized HP gastritis may induce the systemic inflammatory responses and enhance lupus progression in some patients with lupus. Thus patients with dyspepsia or increased systemic cytokine of unknown causes should be further investigated for HP-induced chronic gastritis. Clinical studies to confirm these findings in humans are necessary, which may change our current approach to clinical management of lupus.

## Author Contributions

SS and WP designed and coordinated all the experiments, performed *in vitro* and *in vivo* experiments, and wrote the manuscript and approved. JM, PT, and AT-U performed *in vitro* experiments, and approved the manuscript. JW performed histopathology and approved the manuscript. ST designed experiment and approved the manuscript. AL designed and coordinated all the experiments, analyzed all of these experiment and wrote the manuscript and approved.

## Conflict of Interest Statement

The authors declare that the research was conducted in the absence of any commercial or financial relationships that could be construed as a potential conflict of interest.

## Supplementary Material

FIGURE S1 The representative patterns of capillary protein electrophoresis from wild-type (FcγRIIb +/+) and FcγRIIb-/- mice with phosphate buffer solution (PBS) gavage or *H. pylori* administration were demonstrated **(A–D)**.

## References

[B1] Asahi A. Nishimoto T. Okazaki Y. Suzuki H. Masaoka T. Kawakami Y. (2008). *Helicobacter pylori* eradication shifts monocyte Fcgamma receptor balance toward inhibitory FcgammaRIIB in immune thrombocytopenic purpura patients. *J. Clin. Invest.* 118 2939–2949. 18654664 10.1172/JCI34496 PMC2483681

[B2] Bayraktaroğlu T. Aras A. S. Aydemir S. Davutoğlu C. Üstündağ Y. Atmaca H. (2004). Serum levels of tumor necrosis factor-α, interleukin-6 and interleukin-8 are not increased in dyspeptic patients with *Helicobacter pylori*-associated gastritis. *Mediators Inflamm.* 13 25–28. 10.1080/09629350410001664789 15203561 PMC1781536

[B3] Bolland S. Ravetch J. V. (2000). Spontaneous autoimmune disease in Fc(gamma)RIIB-deficient mice results from strain-specific epistasis. *Immunity* 13 277–285. 10.1016/S1074-7613(00)00027-3 10981970

[B4] Bruhns P. (2012). Properties of mouse and human IgG receptors and their contribution to disease models. *Blood* 119 5640–5649. 10.1182/blood-2012-01-380121 22535666

[B5] Bussiere F. I. Chaturvedi R. Asim M. Hoek K. L. Cheng Y. Gainor J. (2006). Low multiplicity of infection of *Helicobacter pylori* suppresses apoptosis of B lymphocytes. *Cancer Res.* 66 6834–6842. 10.1158/0008-5472.CAN-05-4197 16818661

[B6] Chmiela M. Czkwianianc E. Wadstrom T. Rudnicka W. (1997). Role of *Helicobacter pylori* surface structures in bacterial interaction with macrophages. *Gut* 40 20–24. 10.1136/gut.40.1.20 9155570 PMC1027002

[B7] Chu Z. T. Tsuchiya N. Kyogoku C. Ohashi J. Qian Y. P. Xu S. B. (2004). Association of Fcgamma receptor IIb polymorphism with susceptibility to systemic lupus erythematosus in Chinese: a common susceptibility gene in the Asian populations. *Tissue Antigens* 63 21–27. 10.1111/j.1399-0039.2004.00142.x 14651519

[B8] Clancy C. D. Forde B. M. Moore S. A. O’Toole P. W. (2012). Draft genome sequences of *Helicobacter pylori* strains 17874 and P79. *J. Bacteriol.* 194:2402. 10.1128/JB.00230-12 22493206 PMC3347063

[B9] Clatworthy M. R. Willcocks L. Urban B. Langhorne J. Williams T. N. Peshu N. (2007). Systemic lupus erythematosus-associated defects in the inhibitory receptor FcgammaRIIb reduce susceptibility to malaria. *Proc. Natl. Acad. Sci. U.S.A.* 104 7169–7174. 10.1073/pnas.0608889104 17435165 PMC1855357

[B10] De Filippo K. Henderson R. B. Laschinger M. Hogg N. (2008). Neutrophil chemokines, K C and macrophage-inflammatory protein-2 are newly synthesized by tissue macrophages using distinct TLR signaling pathways. *J. Immunol.* 180 4308–4315. 10.4049/jimmunol.180.6.4308 18322244

[B11] Ding J. Fang Y. Xiang Z. (2015). Antigen/IgG immune complex-primed mucosal mast cells mediate antigen-specific activation of co-cultured T cells. *Immunology* 144 387–394. 10.1111/imm.12379 25196548 PMC4557675

[B12] Dixon M. F. Genta R. M. Yardley J. H. Correa P. (1996). Classification and grading of gastritis. The updated sydney system. International workshop on the histopathology of gastritis, Houston 1994. *Am. J. Surg. Pathol.* 20 1161–1181. 10.1097/00000478-199610000-00001 8827022

[B13] Esposito S. Bosis S. Semino M. Rigante D. (2014). Infections and systemic lupus erythematosus. *Eur. J. Clin. Microbiol. Infect. Dis.* 33 1467–1475. 10.1007/s10096-014-2098-7 24715155

[B14] Ferrero R. L. Ave P. Ndiaye D. Bambou J. C. Huerre M. R. Philpott D. J. (2008). NF-kappaB activation during acute *Helicobacter pylori* infection in mice. *Infect. Immun.* 76 551–561. 10.1128/IAI.01107-07 18070899 PMC2223451

[B15] Floch P. Laur A. M. Korolik V. Chrisment D. Cappellen D. Idrissi Y. (2015). Characterisation of inflammatory processes in *Helicobacter pylori*-induced gastric lymphomagenesis in a mouse model. *Oncotarget* 6 34525–34536. 10.18632/oncotarget.5948 26439692 PMC4741470

[B16] Fujimura K. Kuwana M. Kurata Y. Imamura M. Harada H. Sakamaki H. (2005). Is eradication therapy useful as the first line of treatment in *Helicobacter pylori*-positive idiopathic thrombocytopenic purpura? Analysis of 207 eradicated chronic ITP cases in Japan. *Int. J. Hematol.* 81 162–168. 10.1532/IJH97.04146 15765787

[B17] Giles B. M. Boackle S. A. (2013). Linking complement and anti-dsDNA antibodies in the pathogenesis of systemic lupus erythematosus. *Immunol. Res.* 55 10–21. 10.1007/s12026-012-8345-z 22941560 PMC4018221

[B18] Hasni S. Ippolito A. Illei G. G. (2011). *Helicobacter pylori* and autoimmune diseases. *Oral Dis.* 17 621–627. 10.1111/j.1601-0825.2011.01796.x 21902767 PMC3653166

[B19] He Q. Wang J. P. Osato M. Lachman L. B. (2002). Real-time quantitative PCR for detection of *Helicobacter pylori*. *J. Clin. Microbiol.* 40 3720–3728. 10.1128/JCM.40.10.3720-3728.2002 12354871 PMC130860

[B20] Hooi J. K. Y. Lai W. Y. Ng W. K. Suen M. M. Y. Underwood F. E. Tanyingoh D. (2017). Global prevalence of *Helicobacter pylori* infection: systematic review and meta-analysis. *Gastroenterology* 153 420–429. 10.1053/j.gastro.2017.04.022 28456631

[B21] Hussain K. Hargreaves C. E. Roghanian A. Oldham R. J. Chan H. T. Mockridge C. I. (2015). Upregulation of fcgammaRIIb on monocytes is necessary to promote the superagonist activity of TGN1412. *Blood* 125 102–110. 10.1182/blood-2014-08-593061 25395427

[B22] Jakes R. W. Bae S. C. Louthrenoo W. Mok C. C. Navarra S. V. Kwon N. (2012). Systematic review of the epidemiology of systemic lupus erythematosus in the Asia-Pacific region: prevalence, incidence, clinical features, and mortality. *Arthritis Care Res.* 64 159–168. 10.1002/acr.20683 22052624

[B23] Kaparakis M. Walduck A. K. Price J. D. Pedersen J. S. van Rooijen N. Pearse M. J. (2008). Macrophages are mediators of gastritis in acute *Helicobacter pylori* infection in C57BL/6 mice. *Infect. Immun.* 76 2235–2239. 10.1128/IAI.01481-07 18332213 PMC2346689

[B24] Keep S. Borlace G. Butler R. Brooks D. (2010). Role of immune serum in the killing of *Helicobacter pylori* by macrophages. *Helicobacter* 15 177–183. 10.1111/j.1523-5378.2010.00750.x 20557358

[B25] Khaiboullina S. F. Abdulkhakov S. Khalikova A. Safina D. Martynova E. V. Davidyuk Y. (2016). Serum cytokine signature that discriminates *Helicobacter pylori* positive and negative juvenile gastroduodenitis. *Front. Microbiol.* 7:1916. 10.3389/fmicb.2016.01916 28018296 PMC5156714

[B26] Kodama M. Murakami K. Sato R. Okimoto T. Nishizono A. Fujioka T. (2005). *Helicobacter pylori*-infected animal models are extremely suitable for the investigation of gastric carcinogenesis. *World J. Gastroenterol.* 11 7063–7071. 10.3748/wjg.v11.i45.7063 16437649 PMC4725077

[B27] Konturek P. C. Brzozowski T. Konturek S. J. Stachura J. Karczewska E. Pajdo R. (1999). Mouse model of *Helicobacter pylori* infection: studies of gastric function and ulcer healing. *Aliment Pharmacol. Ther.* 13 333–346. 10.1046/j.1365-2036.1999.00476.x 10102967

[B28] Kuwana M. (2014). *Helicobacter pylori*-associated immune thrombocytopenia: clinical features and pathogenic mechanisms. *World J. Gastroenterol.* 20 714–723. 10.3748/wjg.v20.i3.714 24574745 PMC3921481

[B29] Leelahavanichkul A. Yan Q. Hu X. Eisner C. Huang Y. Chen R. (2010). Angiotensin II overcomes strain-dependent resistance of rapid CKD progression in a new remnant kidney mouse model. *Kidney Int.* 78 1136–1153. 10.1038/ki.2010.287 20736988 PMC3113489

[B30] Mahachai V. Vilaichone R. K. Pittayanon R. Rojborwonwitaya J. Leelakusolvong S. Kositchaiwat C. (2016). Thailand consensus on *Helicobacter pylori* treatment 2015. *Asian Pac. J. Cancer Prev.* 17 2351–2360. 27268597

[B31] Midolo P. Marshall B. J. (2000). Accurate diagnosis of *Helicobacter pylori*. Urease tests. *Gastroenterol. Clin. North Am.* 29 871–878. 10.1016/S0889-8553(05)70154-0 11190071

[B32] Miliukene V. V. Biziuliavichene G. Khaustova L. P. Pilinkene A. V. Biziuliavichius G. A. (2007). [Determination of quantitative parameters of escherichia coli phagocytosis by mouse peritoneal macrophages]. *Tsitologiia* 49 853–857. 10.1134/S1990519X07050112 18074775

[B33] Munoz L. E. Janko C. Schulze C. Schorn C. Sarter K. Schett G. (2010). Autoimmunity and chronic inflammation – two clearance-related steps in the etiopathogenesis of SLE. *Autoimmun. Rev.* 10 38–42. 10.1016/j.autrev.2010.08.015 20817127

[B34] Ondee T. Surawut S. Taratummarat S. Hirankarn N. Palaga T. Pisitkun P. (2017). Fc gamma receptor IIb deficient mice: a lupus model with increased endotoxin tolerance-related sepsis susceptibility. *Shock* 47 743–752. 10.1097/SHK.0000000000000796 27849678

[B35] Podolska M. J. Biermann M. H. Maueroder C. Hahn J. Herrmann M. (2015). Inflammatory etiopathogenesis of systemic lupus erythematosus: an update. *J. Inflamm. Res.* 8 161–171. 26316795 10.2147/JIR.S70325 PMC4548750

[B36] Ravetch J. V. Bolland S. (2001). IgG Fc receptors. *Annu. Rev. Immunol.* 19 275–290. 10.1146/annurev.immunol.19.1.275 11244038

[B37] Rigante D. Esposito S. (2015). Infections and systemic lupus erythematosus: binding or sparring partners? *Int. J. Mol. Sci.* 16 17331–17343 10.3390/ijms160817331 26230690 PMC4581196

[B38] Sawalha A. H. Schmid W. R. Binder S. R. Bacino D. K. Harley J. B. (2004). Association between systemic lupus erythematosus and *Helicobacter pylori* seronegativity. *J. Rheumatol.* 31 1546–1550. 15290733

[B39] Sgouras D. N. Panayotopoulou E. G. Martinez-Gonzalez B. Petraki K. Michopoulos S. Mentis A. (2005). *Lactobacillus johnsonii* La1 attenuates *Helicobacter pylori*-associated gastritis and reduces levels of proinflammatory chemokines in C57BL/6 mice. *Clin. Diagn. Lab. Immunol.* 12 1378–1386. 10.1128/CDLI.12.12.1378-1386.2005 16339060 PMC1317072

[B40] Siriboonrit U. Tsuchiya N. Sirikong M. Kyogoku C. Bejrachandra S. Suthipinittharm P. (2003). Association of Fcgamma receptor IIb and IIIb polymorphisms with susceptibility to systemic lupus erythematosus in thais. *Tissue Antigens* 61 374–383. 10.1034/j.1399-0039.2003.00047.x 12753656

[B41] Skene C. Young A. Every A. Sutton P. (2007). *Helicobacter pylori* flagella: antigenic profile and protective immunity. *FEMS Immunol. Med. Microbiol.* 50 249–256. 10.1111/j.1574-695X.2007.00263.x 17521391

[B42] Smith K. G. Clatworthy M. R. (2010). FcgammaRIIB in autoimmunity and infection: evolutionary and therapeutic implications. *Nat. Rev. Immunol.* 10 328–343. 10.1038/nri2762 20414206 PMC4148599

[B43] Surawut S. Ondee T. Taratummarat S. Palaga T. Pisitkun P. Chindamporn A. (2017). The role of macrophages in the susceptibility of Fc gamma receptor IIb deficient mice to *Cryptococcus neoformans*. *Sci. Rep.* 7:40006. 10.1038/srep40006 28074867 PMC5225418

[B44] Thirumurthi S. Graham D. Y. (2012). *Helicobacter pylori* infection in India from a western perspective. *Indian J. Med. Res.* 136 549–562. 23168695 PMC3516022

[B45] Tsuchiya N. Kyogoku C. (2005). Role of Fc gamma receptor IIb polymorphism in the genetic background of systemic lupus erythematosus: insights from Asia. *Autoimmunity* 38 347–352. 10.1080/08916930500123926 16227149

[B46] Wu Z. Zhou J. Prsoon P. Wei X. Liu X. Peng B. (2012). Low expression of FCGRIIB in macrophages of immune thrombocytopenia-affected individuals. *Int. J. Hematol.* 96 588–593. 10.1007/s12185-012-1187-6 23054650

[B47] Xie C. Lu N. H. (2015). Review: clinical management of *Helicobacter pylori* infection in China. *Helicobacter* 20 1–10. 10.1111/hel.12178 25382801

[B48] Yamanishi S. Iizumi T. Watanabe E. Shimizu M. Kamiya S. Nagata K. (2006). Implications for induction of autoimmunity via activation of B-1 cells by *Helicobacter pylori* urease. *Infect. Immun.* 74 248–256. 10.1128/IAI.74.1.248-256.2006 16368978 PMC1346662

