# Supplementary figures and images for: Helicobacter pylori Infection Increased Anti-dsDNA and Enhanced Lupus Severity in Symptomatic FcγRIIb-Deficient Lupus Mice
Source: Front Microbiol. 2018 Jul 6;9:1488. doi: 10.3389/fmicb.2018.01488 (PMC6043646; doi:10.3389/fmicb.2018.01488)

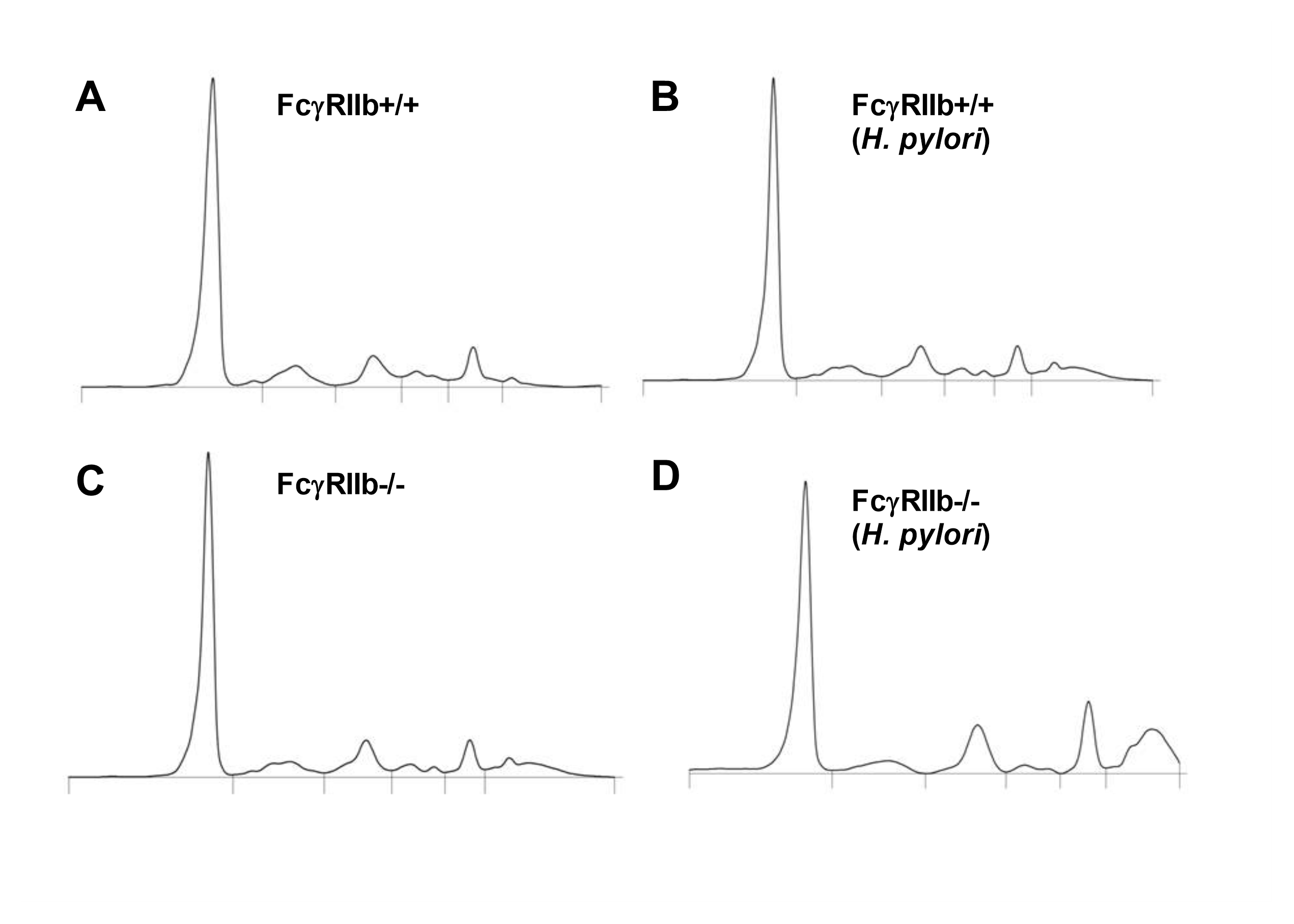

Supplement: FIGURE S1 — The representative patterns of capillary protein electrophoresis from wild-type (FcγRIIb +/+) and FcγRIIb-/- mice with phosphate buffer solution (PBS) gavage or H. pylori administration were demonstrated (A–D). [file Image_1.TIF]
